# Disparate roles of zinc in chemical hypoxia-induced neuronal death

**DOI:** 10.3389/fncel.2015.00001

**Published:** 2015-01-23

**Authors:** Sujeong Kim, Jung-Woo Seo, Shin Bi Oh, So Hee Kim, Inki Kim, Nayoung Suh, Joo-Yong Lee

**Affiliations:** ^1^Asan Institute for Life Sciences, Asan Medical Center, SeoulSouth Korea; ^2^Department of Neurology, University of Ulsan College of Medicine, SeoulSouth Korea

**Keywords:** metal chelation, iron, delayed neuronal death, neuroprotection, brain injury

## Abstract

Accumulating evidence has provided a causative role of zinc (Zn^2+^) in neuronal death following ischemic brain injury. Using a hypoxia model of primary cultured cortical neurons with hypoxia-inducing chemicals, cobalt chloride (1 mM CoCl_2_), deferoxamine (3 mM DFX), and sodium azide (2 mM NaN_3_), we evaluated whether Zn^2+^ is involved in hypoxic neuronal death. The hypoxic chemicals rapidly elicited intracellular Zn^2+^ release/accumulation in viable neurons. The immediate addition of the Zn^2+^ chelator, CaEDTA or N,N,N’N’-tetrakis-(2-pyridylmethyl) ethylenediamine (TPEN), prevented the intracellular Zn^2+^ load and CoCl_2_-induced neuronal death, but neither 3 hour later Zn^2+^ chelation nor a non-Zn^2+^ chelator ZnEDTA (1 mM) demonstrated any effects. However, neither CaEDTA nor TPEN rescued neurons from cell death following DFX- or NaN_3_-induced hypoxia, whereas ZnEDTA rendered them resistant to the hypoxic injury. Instead, the immediate supplementation of Zn^2+^ rescued DFX- and NaN_3_-induced neuronal death. The iron supplementation also afforded neuroprotection against DFX-induced hypoxic injury. Thus, although intracellular Zn^2+^ release/accumulation is common during chemical hypoxia, Zn^2+^ might differently influence the subsequent fate of neurons; it appears to play a neurotoxic or neuroprotective role depending on the hypoxic chemical used. These results also suggest that different hypoxic chemicals may induce neuronal death via distinct mechanisms.

## Introduction

Zinc (Zn^2+^) contributes to neuronal injury according to various experimental models of excitotoxic brain injury (Sensi et al., 2011). Exposing cortical cultures to high levels of Zn^2+^ induces extensive neuronal and glial cell death (Choi et al., 1988). The intracellular release of Zn^2+^ subsequent to exposure to oxidative or nitrosative agents leads to neuronal degeneration in cultured neurons (Bossy-Wetzel et al., 2004; Hwang et al., 2008). In animal models of acute brain injury, including cerebral ischemia, epilepsy, and trauma, a large accumulation of Zn^2+^ occurs in degenerating neurons as demonstrated by the Zn^2+^-specific fluorescence dyes (Frederickson et al., 1988; Tønder et al., 1990; Koh et al., 1996; Lee et al., 2000; Suh et al., 2000). Intracellular Zn^2+^ release/accumulation obviously precedes neuronal death in these experimental models (Koh et al., 1996) since Zn^2+^ chelators, such as ethylenediaminetetraacetic acid (EDTA; Koh et al., 1996; Lee et al., 2000; Frederickson et al., 2002; Suh et al., 2004) and N,N,N′,N′,-tetrakis-(2-pyridylmethyl)-ethylenediamine (TPEN; Bossy-Wetzel et al., 2004; Cho et al., 2010), intercept intracellular Zn^2+^ load to suppress neuronal death.

Cerebral hypoxia develops when the brain suffers from oxygen shortage due to the blockage of blood flow, resulting in extensive neuronal death in selective vulnerable areas (Sharp and Bernaudin, 2004). Since the involvement of Zn^2+^ in neuronal death in the hippocampal CA1 area following transient global cerebral ischemia was reported (Koh et al., 1996), studies have suggested that excessive Zn^2+^ release/accumulation leads to neuronal injury after hypoxia/ischemia (Sensi et al., 2011). When mouse hippocampal slices are subjected to oxygen and glucose deprivation (OGD)—which is a typical experimental model of hypoxia/ischemia—intracellular Zn^2+^ becomes prominent in degenerating neurons, whereby the Zn^2+^ chelator CaEDTA attenuates both Zn^2+^ accumulation and neuronal death (Yin et al., 2002; Medvedeva et al., 2009). Similarly, hypobaric hypoxia causes Zn^2+^-mediated inflammation and apoptosis in neurons of the mouse hippocampus, which are also reversed by CaEDTA (Malairaman et al., 2014). Recent studies have provided that Zn^2+^ promotes hypoxic cell death by upregulating hypoxia-inducible transcription factor-1α (HIF1α) via an activation of NADPH oxidase or poly(ADP-ribose) polymerase (PARP; Pan et al., 2013; Malairaman et al., 2014).

While the precise control of oxygen level is crucial to simulate hypoxic condition in cell culture, it is difficult, so various *in vitro* models of neuronal hypoxia have been provided containing OGD models. Some divalent cations such as cobalt (Co^2+^), nickel (Ni^2+^), and the iron-chelator deferoxamine (DFX), have been applicable to mimic hypoxic conditions in cultured cells because they activate hypoxic signals by stabilizing the expression of HIF1α (Ho and Bunn, 1996). Sodium azide (NaN_3_) and potassium cyanide (KCN) are also potent inhibitors of cytochrome c oxidase (i.e., complex IV of the mitochondrial respiratory chain) to induce chemical hypoxia (Roemgens et al., 2011). However, although the hypoxic chemicals have helped us to understand the molecular events that underlie the hypoxic neuronal death, it remains unclear whether chemical hypoxia also involves Zn^2+^-mediated neuronal injury in cultured neurons.

In this study, we found that intracellular Zn^2+^ release/accumulation occurs in primary neuronal cells shortly after exposure to CoCl_2_, DFX, or NaN_3_, whereas the effects of Zn^2+^ chelation on neuronal fate differ depending on the hypoxia-inducing chemicals used. This study shows the disparate roles of Zn^2+^ in neuronal death following chemical hypoxia.

## Materials and methods

### Primary cortical neuron cultures

We used ICR mice in this study, in accordance with the Guidelines of the Asan Institute for Life Sciences for the Care and Use of Laboratory Animals. Cerebral cortical tissues were dissected from the brains of fetal ICR mice (Koatech, Pyeongtaek, Korea) at embryonic day E14, dissociated in Ca^2+^/Mg^2+^-free Hank’s balanced salt solution (HBSS; Invitrogen, Carlsbad, CA, USA) containing 0.25% trypsin-EDTA (Invitrogen), and filtered through 40-µm nylon cell strainer (BD Biosciences, Durham, NC, USA). Cells were washed in Dulbecco’s modified Eagle’s medium (DMEM; Invitrogen) with 10% fetal bovine serum (FBS; Gibco, Grand Island, NY, USA) and penicillin/streptomycin (Invitrogen), and resuspended in serum-free Neurobasal medium (Invitrogen) containing the B27 supplement (Invitrogen), L-glutamine (2 mM; Invitrogen) and penicillin/streptomycin. Cells were plated at a density of 5 × 10^5^–10^6^ cells/well on poly-L-lysine-coated well culture dishes and grown in a humidified 5% CO_2_ incubator at 37°C. Cultures were treated with cytosine arabinoside (Ara-C, 2 µM; Sigma, St. Louis, MO, USA) for 24 h at 3 days *in vitro* (DIV3) to halt the growth of non-neuronal cells, and maintained in fresh Neurobasal medium with B27 until used in experiments between DIV10–11.

### Induction of chemical hypoxia

All chemicals used in this study, except CoEDTA (TCI, Tokyo, Japan), were purchased from Sigma-Aldrich or Fluka (St. Louis, MO, USA).

To induce chemical hypoxia, cells were treated with CoCl_2_ (1 mM) (Fang et al., 2008; Zhang et al., 2011) or DFX (3 mM) (Almli et al., 2001; Guelman et al., 2004) for 2 h, or NaN_3_ (2 mM) for 1 h (Garnier et al., 2003; Selvatici et al., 2009) in glucose-free MEM, and then the media was freshly replaced. To define the roles of the intracellular metals in neurons during chemical hypoxia, we added the metal chelator EDTA with various salts (CaEDTA, ZnEDTA, CoEDTA, or FeEDTA), or TPEN to the media at 10 min or 3 h after hypoxic chemical treatment.

### Cell viability assessment

Cell viability was determined using the 3-(4,5-dimethylthiazol-2-yl)-2,5-diphenyltetrazolium bromide (MTT) assay (Stanciu et al., 2000; White et al., 2001). Cortical neurons were grown on poly-L-lysine coated 24-well plates, and treated with MTT (final concentration, 0.5 mg/mL in culture media; Amresco, Solon, OH, USA) at 37°C for 2 h. After culture medium was completely removed, the insoluble formazan crystals were dissolved in dimethyl sulfoxide (DMSO; 200 µL). The reaction products (in 100 µL aliquots) were measured at 570 nm using a microplate reader (Synergy H1 Hybrid; BioTek Instruments, Winooski, VT, USA). All experiments were consisted of at least three independent repeats, and each experiment contained three parallel cultures. Duplicate measurements of MTT absorbance were performed for each sample. Resultantly, percentage of viable cells in drug-treated cultures was determined relative to vehicle-treated control cells.

In addition, neuronal cell death was visually detected by staining the nuclei with Hoechst 33342 or propidium iodide (PI). After the cells were incubated in the presence of Hoechst 33342 (10 µg/mL; Invitrogen) and PI (1 µg/mL; Sigma) for 15 min, the fluorescent phenotypes of the nuclei were examined under a fluorescence inverted microscope (Axio Observer.Z1; Carl Zeiss, Göttingen, Germany) using a DAPI filter (beam splitter, 395 nm; excitation, 365 ± 50 nm; emission, 445 ± 50 nm) and a Set20 filter (beam splitter, 560 nm; excitation, 546 ± 12 nm; emission, 575–640 nm), respectively. PI-fluorescent red nuclei-containing neurons were considered dead or dying as the dye is excluded by viable cells.

### Detection and measurement of intracellular Zn^2+^

To assess the levels of intracellular Zn^2+^, cells were incubated with 2 µM FluoZin-3 AM (Kd for Zn^2+^, about 15 nM) (Molecular Probes, Eugene, OR, USA) for 30 min and washed with fresh culture medium (Gee et al., 2002). FluoZin-3 reactive cells were examined or photographed under consistent imaging conditions with an inverted fluorescence microscope (Axio Observer.Z1) using a FITC filter (beam splitter, 495 nm; excitation, 450–490 nm; emission, 500–550 nm) equipped with self-adjusting lamps and an AxioCam digital camera (Carl Zeiss).

To quantify the level of intracellular Zn^2+^, we took the photographs (magnification, 100×) from three spots randomly selected from each culture well, and measured the mean intensity of FluoZin-3-fluorescence in neurons using ImagePro Plus software (Media Cybernetics, Silver Spring, MD, USA). After subtracting the background intensity (which was determined by assessing areas without cells), the average intensity of FluoZin-3-fluorescence per neuron was reported as the level of intracellular Zn^2+^.

### Statistical analysis

Values were expressed as the mean ± standard errors of mean (SEM). Statistical comparisons were performed using one-way analysis of variance (ANOVA) followed by the *post hoc* Student–Newman–Keuls test using GraphPad InStat (GraphPad Software, La Jolla, CA, USA). *P* values < 0.05 were considered to indicate statistical significance.

## Results

Since an early study implicated Zn^2+^ in neuronal death following transient global cerebral ischemia in rats (Koh et al., 1996), a large body of evidence has attributed excitotoxic neuronal injury to Zn^2+^ overload in neurons (Sensi et al., 2011). This is principally based on the proof-of-concept that intracellular Zn^2+^ overload occurs in degenerating neurons (correlation) before death (precedence), and that such pathological phenomena are eliminated when Zn^2+^ is chelated or removed (interference) (Koh et al., 1996). However, to our knowledge, there are a few study regarding that intracellular Zn^2+^ indeed takes part in hypoxic neuronal death, except experiments in which cerebral organ cultures (but not neuronal cells) were exclusively subjected to OGD conditions (Büchner et al., 2002; Yin et al., 2002; Miyawaki et al., 2004; Medvedeva et al., 2009). Thus, we first performed our current study to determine if Zn^2+^ release/accumulation occurs in association with neuronal death in primary neuronal cultures exposed to a hypoxic chemical, CoCl_2_, DFX, or NaN_3_.

### Intracellular Zn^2+^ release/accumulation in neurons exposed to hypoxic chemicals

**Figure 1:**
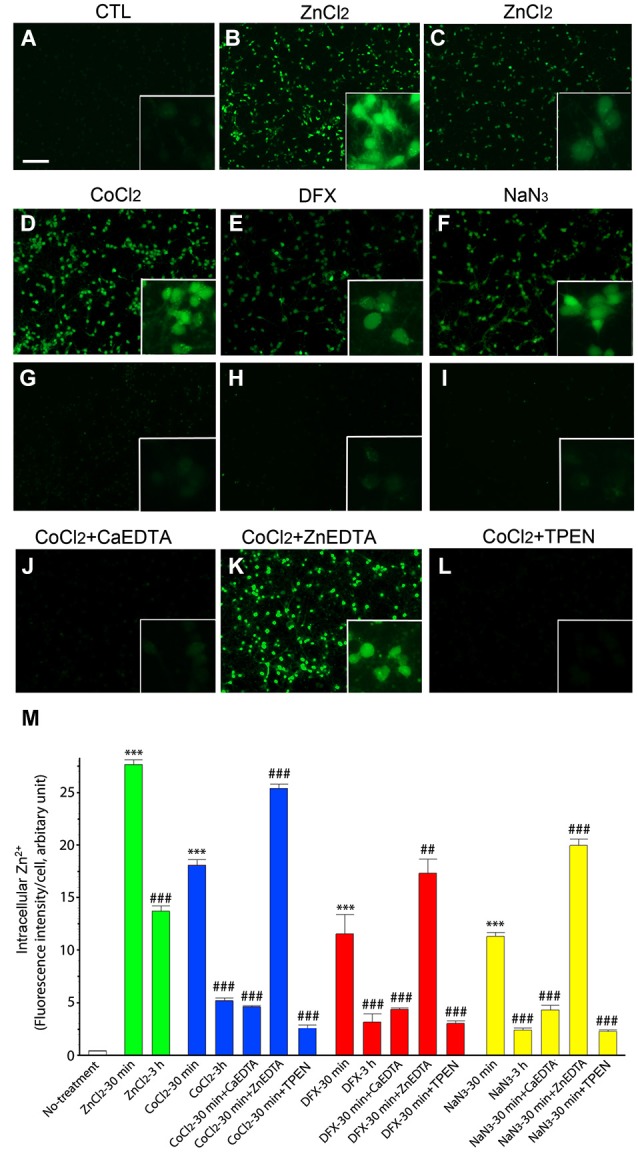
**Intracellular Zn^2+^ release/accumulation in neurons exposed to hypoxia-inducing chemicals**. At 30 min **(A,B,D–F)** or 3 h **(C,G–I)** after treatment with 200 µM ZnCl_2_ **(B,C)**, 1 mM CoCl_2_ **(D,G)**, 3 mM DFX **(E,H)**, or 2 mM NaN_3_ **(F,I)**, cultured neurons were incubated with 2 µM FluoZin-3 AM for 30 min. CoCl_2_-exposed neurons were also added with 1 mM CaEDTA **(J)**, 1 mM ZnEDTA **(K)** or TPEN (0.5 µM; **L**) 10 min later. Insets show highly magnified FluoZin-3–stained neurons. Scale bar, 100 µm. The intensity of FluoZin-3 fluorescence was quantified in neuronal cultures under a variety of chemical combinations **(M)**. Bars denote the mean intensity of the intracellular Zn^2+^ fluorescence per neuron after subtracting the background level taken from the cell-free area. All experiments were performed in four independent replications (*n* = 4), and the intensity of FluoZin-3 fluorescence was quantified by measuring 3 random spots per each independent sample and expressed as arbitrary units. Data are the mean ± SEM of quadruplicate experiments. ^##^ < 0.01, or *** or ^###^*p* < 0.001 in comparison with the corresponding control according to one-way ANOVA followed by the Student–Newman–Keuls *post hoc* test.

Intracellular Zn^2+^ was detected using the Zn^2+^-specific fluorescent indicator FluoZin-3 (Gee et al., 2002), which reacted with Zn^2+^ to emit bright green fluorescence in cortical neurons 30 min after the addition of 200 µM ZnCl_2_ (Figures 1B,M). Three hours later, FluoZin-3-fluorescence was significantly attenuated in the ZnCl_2_-treated neuron cultures (Figures 1C,M).

Similarly, we noted the rapid evolution of FluoZin-3-fluorescence in neurons following 30 min-exposures to the hypoxia-inducing chemicals CoCl_2_ (1 mM; Figures 1D,M), DFX (3 mM; Figures 1E,M), and NaN_3_ (2 mM; Figures 1F,M). Three hours later, the intensity of the intracellular fluorescence was significantly reduced in the hypoxic chemical-treated neurons (Figures 1G–I,M). These results thus indicate that Zn^2+^ was released and accumulated in neurons shortly after exposure to hypoxic chemicals, and thereafter gradually disappears as the time progresses.

### Intracellular Zn^2+^ release/accumulation precedes neuronal death during chemical hypoxia

**Figure 2:**
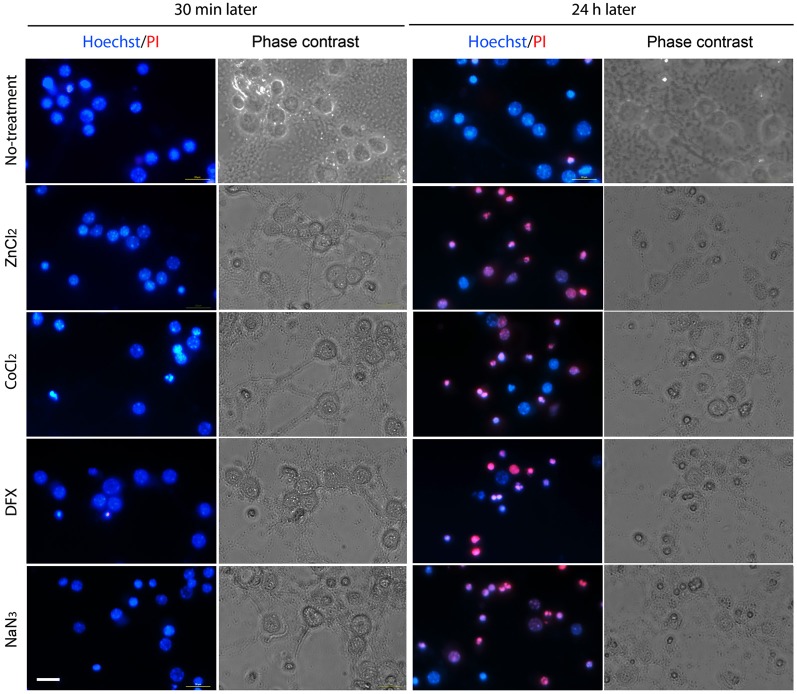
**Morphological determination of chemical hypoxia-induced neuronal death**. Neuronal cultures were photographed in the absence (2nd and 4th columns) or presence (1st and 3rd columns) of double staining with Hoechst 33342 (blue) and propidium iodide (PI; red) at 30 min (1st and 2nd columns) or 24 h (3rd and 4th columns) after non-treatment (1st row) or exposure to 1 mM CoCl_2_ (2nd row), 3 mM DFX (3rd row) or 2 mM NaN_3_ (4th row). Scale bar, 20 µm.

**Figure 3:**
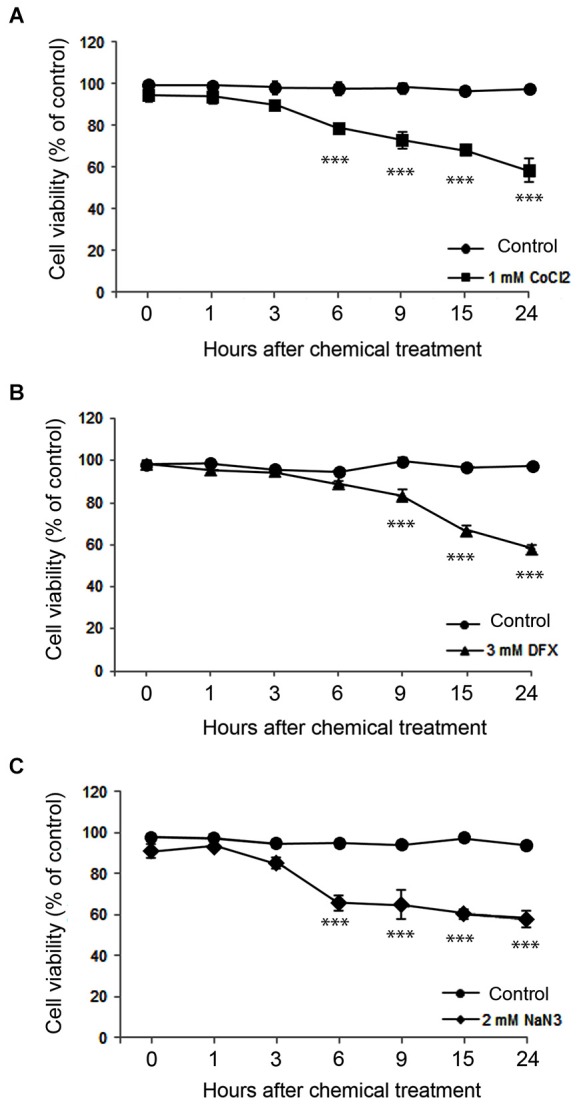
**Time course of neuronal death assessed using the MTT cell viability assay at various time points after 1 mM CoCl_2_- (A), 3 mM DFX- (B), or 2 mM NaN_3_- (C) treatment**. Data are the mean ± SEM of quadruplicate independent experiments, which contained three parallel cultures. ***p < 0.001 in comparison with the corresponding control treatment according to one-way ANOVA followed by the Student–Newman–Keuls *post hoc* test.

We assessed neuronal death for 24 h after hypoxic insult. On the basis of PI exclusion assay, we found that neurons were still intact at 30 min after ZnCl_2_- or hypoxic chemical treatment (Figure 2), when Zn^2+^ had highly accumulated in neurons (Figure 1). However, neuronal death began to appear about 3 h later (Figure 3) when intracellular Zn^2+^-fluorescence decreased (Figure 1), and gradually increased as time progressed, a phenomenon that was further evidenced by MTT cell viability assay (Figure 3). Hence, these data indicate that intracellular Zn^2+^ release/accumulation precedes neuronal death after chemical-induced hypoxia, thus providing the Zn^2+^-induced delayed neuronal death.

### Effects of Zn^2+^ chelation on chemical hypoxia-induced neuronal death

To relieve intracellular Zn^2+^ overload, we added CaEDTA or ZnEDTA (each 1 mM) to the media at 10 min after exposure of the neurons to ZnCl_2_ or hypoxic chemicals, and examined FluoZin-3-fluorescence 30 min later. Consistent with our expectations that EDTA would fully chelate intracellular Zn^2+^ (Frederickson et al., 2002), CaEDTA reduced FluoZin3-fluorescence in the ZnCl_2_- or hypoxic chemical-exposed neurons (Figure 1) whereas ZnEDTA increased fluorescence (Figures 1K,M). Furthermore, TPEN (0.5 µM) perfectly depleted it (Figures 1L,M).

**Figure 4:**
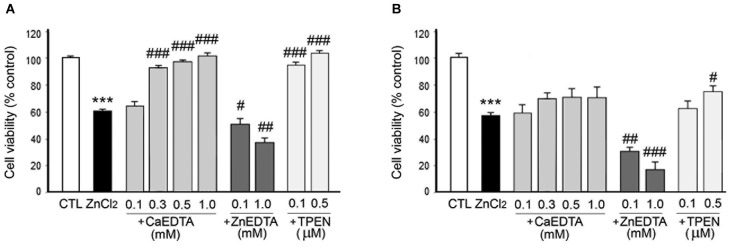
**Effects of metal chelation on ZnCl_2_-induced neuron death. ZnCl_2_ (200 µM)-exposed neurons were followed by the immediate (A) or 3 h later (B) addition of CaEDTA (0.1–1.0 mM), ZnEDTA (0.1 or 1.0 mM), or TPEN (0.1 or 0.5 µM)**. Twenty-four hours after ZnCl_2_ application, neuronal death was assessed by the MTT cell viability assay. Bars denote the mean ± SEM of at least three independent experiments, which each consisted of three parallel cultures. Values were expressed as percentages of non-treated control cells. ^#^*p* < 0.05, ^##^*p* < 0.01, or *** or ^###^*p* < 0.001 in the corresponding comparison according to one-way ANOVA followed by the Student–Newman–Keuls *post hoc* test.

**Figure 5:**
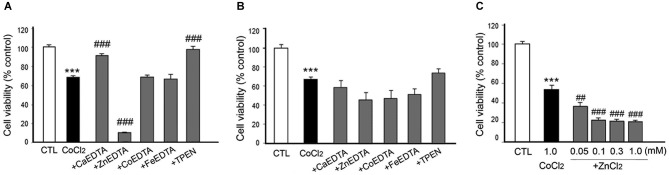
**Effects of metal chelation or Zn^2+^ supplementation on CoCl_2_-induced neuronal death**. CoCl_2_ (1 mM)-treated neurons were followed by the immediate **(A)** or 3 h later **(B)** addition of CaEDTA (1.0 mM), ZnEDTA (1.0 mM), CoEDTA (1.0 mM), FeEDTA (1.0 mM), or TPEN (0.5 µM), or by the immediate addition of ZnCl_2_ (0.05–1.0 mM) **(C)**. Twenty-four hours after CoCl_2_ application, neuronal death was assessed using the MTT cell viability assay. Bars denote the mean ± SEM of at least three independent experiments, which each consisted of three parallel cultures. Values were expressed as percentages of non-treated control cells. ^##^*p* < 0.01, or *** or ^###^*p* < 0.001 in the corresponding comparison by the one-way ANOVA and the Student–Newman–Keuls *post hoc* test.

**Figure 6:**
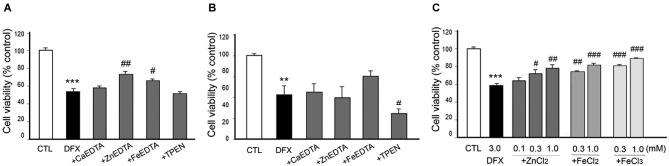
**Effect of metal chelation or supplementation on DFX-induced neuronal death**. DFX (3 mM)-exposed neurons were followed by the immediate **(A)** or 3 h later **(B)** addition of CaEDTA (1.0 mM), ZnEDTA (1.0 mM), FeEDTA (1.0 mM), or TPEN (0.5 µM), or by the immediate addition of ZnCl_2_ (0.1–1.0 mM), FeCl_2_ (0.3 or 1.0 mM) or FeCl_3_ (0.3 or 1.0 mM) **(C)**. Twenty-four hours after DFX exposure, neuronal death was assessed by the MTT cell viability assay. Bars denote the mean ± SEM of at least three independent experiments, which each consisted of three parallel cultures. Values were expressed as percentages of non-treated control cells. ^#^*p* < 0.05, ** or ^##^*p* < 0.01, or *** or ^###^*p* < 0.001 in the corresponding comparison by the one-way ANOVA and the Student–Newman–Keuls *post hoc* test.

**Figure 7:**
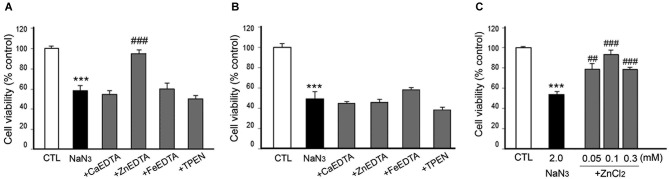
**Effects of metal chelation or Zn^2+^ supplementation on NaN_3_-induced neuron death**. NaN_3_ (2 mM)-exposed neurons were followed by the immediate **(A)** or 3 h later **(B)** addition of CaEDTA (1.0 mM), ZnEDTA (1.0 mM), FeEDTA (1.0 mM), or TPEN (0.5 µM), or by the immediate addition of ZnCl_2_ (0.05–1.0 mM) **(C)**. Twenty-four hours after NaN_3_ application, neuronal death was assessed using the MTT cell viability assay. Bars denote the mean ± SEM of at least three independent experiments, which each consisted of three parallel cultures. Values were expressed as percentages of non-treated control cells. ^#^*p* < 0.05, ** or ^##^*p* < 0.01, or *** or ^###^*p* < 0.001 between the corresponding comparisons by the one-way ANOVA and the Student–Newman–Keuls *post hoc* test.

Previous studies using various experimental models of neurological disease have reported that CaEDTA (but not ZnEDTA) efficiently blocks neuronal death (Koh et al., 1996). Hence, in order to define the Zn^2+^-specific actions on chemical hypoxia-induced neuronal death, we added various salt forms of EDTA (0.1–1.0 mM) or TPEN (0.1–0.5 µM) to the culture media at 10 min or 3 h after exposure to ZnCl_2_ or a hypoxic chemical, and then determined cell viability using the MTT assay (Figures 4–7).

In consistency with the previous studies (Koh et al., 1996), either CaEDTA (0.3–1.0 mM) or TPEN (0.1–0.5 µM) evidently reduced neuronal death when added at 10 min after the ZnCl_2_-exposure (Figure 4A), but the neuroprotective effect was less evident with the 3 h-delayed CaEDTA treatment (Figure 4B). In contrast, ZnEDTA (0.1 or 1.0 mM) aggravated the ZnCl_2_-induced neuronal death (Figures 4A,B). These results support the evidence for Zn^2+^-induced delayed neuronal death.

When either CaEDTA (1 mM) or TPEN (0.5 µM) was added to CoCl_2_-exposed neurons 10 min later, it significantly decreased neuronal death; however, ZnEDTA (1 mM) resulted in severe toxicity, and CoEDTA (1 mM) or FeEDTA (1 mM) had no effect (Figure 5A). Three hour post-treatment of CaEDTA or TPEN rarely affected CoCl_2_-induced neuronal death (Figure 5B). These findings suggest that intracellular Zn^2+^ can cause delayed neuronal death during CoCl_2_-induced hypoxia.

However, it was unexpected that ZnEDTA reduced the DFX- or NaN_3_-induced neuronal death when added 10 min later, but neither CaEDTA nor TPEN (Figures 6A, 7A). FeEDTA also provided some protective effects against DFX-induced hypoxic death (Figure 6A). Three hour post-treatment of EDTA or TPEN had no effect on NaN3 (Figure 6B)- or DFX (Figure 7B)-induced neuronal death.

### Neuroprotective effect of metals in chemical hypoxia

Because ZnEDTA prominently reduced neuronal death following DFX- or NaN_3_-induced hypoxia (Figures 6A, 7A), we investigated whether Zn^2+^ enables neurons to survive the chemical-induced hypoxic damage (Figures 5C, 6C, 7C). As expected, ZnCl_2_ (0.05–1.0 mM) significantly increased the level of CoCl_2_–induced neuronal death (Figure 5C). However, ZnCl_2_ produced protective effects against neuronal death following DFX- or NaN_3_-induced chemical hypoxia (Figures 6C, 7C). Similar to the neuroprotection by FeEDTA against DFX-induced neuronal death (Figure 6A), supplementation with iron (0.3–1.0 mM FeCl_2_ or FeCl_3_) rendered neurons significantly more resistant to DFX-induced chemical hypoxia (Figure 6C). Therefore, apart from CoCl_2_-induced neuronal death that was aggravated by ZnCl_2_, Zn^2+^ is likely to protect neurons against DFX- or NaN_3_-induced chemical hypoxia. Plus, iron (Fe^2+^ or Fe^3+^) may also provide neuroprotective effects against DFX-induced hypoxia (Figure 6C).

## Discussion

The mechanism underlying chemical hypoxia remains unclear. A line of studies have noted to the involvement of iron in stabilizing HIF1α and thereby activating hypoxic signals (Ho and Bunn, 1996). Because HIF1α is rapidly degraded by the polyubiquitination and proteasome pathway, which is manipulated by prolyl-4-hydroxylases (PHDs), it is normally present in cells at low levels (Bruick and McKnight, 2001; Epstein et al., 2001). PHDs essentially require oxygen and iron for their activity, so the depletion of iron from cells could inhibit the activity of the PHDs to stabilize HIF1α from degradation, stimulating the hypoxic responses similar to that observed due to an oxygen shortage (Bruick and McKnight, 2001; Guo et al., 2001). Transition metals (e.g., Co^2+^ or Ni^2+^) and iron chelators (e.g., DFX) could induce hypoxic responses by inhibiting PHD activity via iron replacement or depletion, respectively (Schofield and Ratcliffe, 2004; Choi et al., 2006). Although Zn^2+^ could be another effective replacement metal for iron in PHDs (Shibayama et al., 1986), there have been disputes regarding the roles of Zn^2+^ in hypoxia. Zn^2+^ has recently been found to elevate the intracellular expression of HIF1α through the activation of NADPH oxidase or poly(ADP ribose) polymerase (PARP; Pan et al., 2013; Malairaman et al., 2014). By contrast, Zn^2+^ also inhibits HIF1α activity and the activation of the hypoxia-inducible genes to block the hypoxic responses (Chun et al., 2000, 2001). Thus, while these HIF1α-modulating metal signals may suggest a mechanism of chemical hypoxia, it still remains to be defined how hypoxic chemicals induce neuron death, particularly via intracellular Zn^2+^ release/accumulation.

In this study, when the neuronal cultures were exposed to ZnCl_2_, or the hypoxic chemical CoCl_2_, DFX, or NaN_3_, we observed the intense emission of Zn^2+^-specific FluoZin-3-fluorescence in neurons. To confirm the intracellular Zn^2+^ release/accumulation, we examined that the Zn^2+^-chelator CaEDTA (Koh et al., 1996; Frederickson et al., 2002) evidently eliminated FluoZin-3-fluorescence from the chemical-treated cultures at the higher concentration (1 mM), despite concern that low concentration of CaEDTA perturb no response of FluoZin-3 to Zn^2+^ (Zhao et al., 2008). Moreover, TPEN (0.5 µM) also perfectly depleted FluoZin-3-fluorescence, but the non-Zn^2+^ chelator ZnEDTA (1 mM) (Koh et al., 1996) showed no attenuation of the fluorescence intensity. Therefore, these findings support that Zn^2+^ is robustly released and accumulated in cultured neurons shortly after the hypoxic chemical treatment. A variety of sources of releasable Zn^2+^ has been found in neurons, such as Zn^2+^-bound proteins (Aizenman et al., 2000; Lee et al., 2000, 2003) or Zn^2+^-containing organelles including mitochondria (Jiang et al., 2001; Sensi et al., 2002) or lysosomes (Hwang et al., 2008). In addition, since neurons survived the moment of the highest intracellular Zn^2+^ accumulation and then started to die along with its gradual loss, we guess that Zn^2+^ could cause delayed neuronal death in hypoxic chemical-treated cultures.

However, the effects of Zn^2+^ chelation on chemical hypoxia-induced neuronal death differed depending on the hypoxic chemical that was used. When EDTA was added immediately after CoCl_2_-induced hypoxia, CaEDTA evidently alleviated neuron death, but ZnEDTA potently augmented cell death. However, 3 h delayed CaEDTA rarely reduced CoCl_2_-induced neuronal death. CoEDTA or FeEDTA had no effects. A strong intracellular Zn^2+^-chelator TPEN also produced the neuroprotective effects. These results were comparable to the effect of CaEDTA or TPEN on ZnCl_2_-induced delayed neuronal death, where the immediate Zn^2+^ chelation with CaEDTA or TPEN counteracted the neuronal death but the late CaEDTA showed no protection. It appears that the late Zn^2+^ chelation couldn’t afford to block the death signaling process that has been already triggered by the precedent Zn^2+^ overload in neurons. Therefore, we believe that CoCl_2_-induced hypoxia rapidly triggers intracellular Zn^2+^ release, leading to Zn^2+^ overload in neurons and thereby causing their death. In contrast, there was an opposite case during DFX- or NaN_3_-induced hypoxia. ZnEDTA rather protected neurons from DFX- and NaN_3_-induced hypoxic death, but CaEDTA had no effect. Zn^2+^ supplementation also enabled neurons to survive DFX- or NaN_3_-induced hypoxic damages. These results suggest that Zn^2+^ may be neurotoxic or neuroprotective in neurons during chemical hypoxia; Zn^2+^ may directly cause hypoxic neuronal death (in CoCl_2_-induced hypoxia), or normally participate in neuronal survival or viability (in DFX- or NaN_3_-induced hypoxia). In addition, we found that iron supplementation (Fe^2+^ or Fe^3+^) can protect neurons from DFX-induced hypoxic damage, consistent with speculation that it may make up for DFX-induced iron depletion. However, it is unfortunate that there is no current explanation or information concerning how or why Zn^2+^ plays in the opposite roles in the chemical hypoxia-induced neuronal death.

It is well established that cytosolic calcium (Ca^2+^) overload triggers signal pathways to execute neuronal degeneration after hypoxic/ischemic insult (Lipton, 1999; Bano and Nicotera, 2007; Mattson, 2007; Berna-Erro et al., 2009). Our results might exclude the causative roles of Ca^2+^ in CoCl_2_-induced hypoxic neuronal death due to the evidence consistent with the earlier study (Koh et al., 1996), in which cytosolic Zn^2+^ overload preceded neuronal death and Zn^2+^-specific chelation CaEDTA (but not ZnEDTA) recovered it. Instead, as we failed to determine whether Zn^2+^-specific chelation inhibits neuronal death after DFX- or NaN_3_-inducrd hypoxia, we couldn’t rule out the possibility that Ca^2+^-induced excitotoxicity may contribute to hypoxic neuronal death (Koh et al., 1996; Lipton, 1999; Bano and Nicotera, 2007; Mattson, 2007; Berna-Erro et al., 2009).

In conclusion, we for the first time provide evidence that hypoxia stimulates the intracellular release/accumulation of Zn^2+^ in neurons, and thereby it may contribute to neuronal death or survival. The opposite roles of Zn^2+^ in hypoxic chemical-induced neuron death may not only indicate that different hypoxic chemicals induce neuron death via distinct mechanisms, but reflect the diverse groups of signals that essentially require Zn^2+^ for their functions. Otherwise, Zn^2+^-regulated neuronal fate may be differentially determined depending on the actual range of intracellular Zn^2+^ levels (Cho et al., 2010). To date, chelation study using EDTA or TPEN has focused mainly on the negative roles of Zn^2+^ as a main cause of neuronal death in the context of excitotoxic acute brain injury (Sensi et al., 2011). Instead, this study offers the insight into the positive aspect of Zn^2+^ that it could mediate neuronal survival under such neurological diseases. Further study will be warranted to elucidate the mechanism by which Zn^2+^ enable neurons to survive a variety of neurotoxic circumstances.

## Author contributions

Sujeong Kim and Jung-Woo Seo designed the culture experiments and performed the MTT viability analysis. Shin Bi Oh and So Hee Kim photographed the cultured neurons and performed the image-analysis. Inki Kim and Nayoung Suh managed and discussed the overall study, analyzed the data and prepared the manuscript draft. Joo-Yong Lee conceived and designed the work, approved the data analysis and interpretations, and finally completed the manuscript. All authors saw and approved the completion of the work.

## Conflict of interest statement

The authors declare that the research was conducted in the absence of any commercial or financial relationships that could be construed as a potential conflict of interest.
